# Evaluation of Semiautomatic and Deep Learning–Based Fully Automatic Segmentation Methods on [^18^F]FDG PET/CT Images from Patients with Lymphoma: Influence on Tumor Characterization

**DOI:** 10.1007/s10278-023-00823-y

**Published:** 2023-04-14

**Authors:** Cláudia S. Constantino, Sónia Leocádio, Francisco P. M. Oliveira, Mariana Silva, Carla Oliveira, Joana C. Castanheira, Ângelo Silva, Sofia Vaz, Ricardo Teixeira, Manuel Neves, Paulo Lúcio, Cristina João, Durval C. Costa

**Affiliations:** 1 Nuclear Medicine – Radiopharmacology Department, Champalimaud Foundation, Av. Brasília, 1400-038 Lisbon, Portugal; 2 Hematology Department, Champalimaud Foundation, Av. Brasília, 1400-038 Lisbon, Portugal

**Keywords:** Lymphoma, [^18^F]FDG PET/CT, Computer-assisted image analysis, Reproducibility of results, Artificial intelligence

## Abstract

The objective is to assess the performance of seven semiautomatic and two fully automatic segmentation methods on [^18^F]FDG PET/CT lymphoma images and evaluate their influence on tumor quantification. All lymphoma lesions identified in 65 whole-body [^18^F]FDG PET/CT staging images were segmented by two experienced observers using manual and semiautomatic methods. Semiautomatic segmentation using absolute and relative thresholds, *k*-means and Bayesian clustering, and a self-adaptive configuration (SAC) of *k*-means and Bayesian was applied. Three state-of-the-art deep learning–based segmentations methods using a 3D U-Net architecture were also applied. One was semiautomatic and two were fully automatic, of which one is publicly available. Dice coefficient (DC) measured segmentation overlap, considering manual segmentation the ground truth. Lymphoma lesions were characterized by 31 features. Intraclass correlation coefficient (ICC) assessed features agreement between different segmentation methods. Nine hundred twenty [^18^F]FDG-avid lesions were identified. The SAC Bayesian method achieved the highest median intra-observer DC (0.87). Inter-observers’ DC was higher for SAC Bayesian than manual segmentation (0.94 vs 0.84, *p* < 0.001). Semiautomatic deep learning–based median DC was promising (0.83 (Obs1), 0.79 (Obs2)). Threshold-based methods and publicly available 3D U-Net gave poorer results (0.56 ≤ DC ≤ 0.68). Maximum, mean, and peak standardized uptake values, metabolic tumor volume, and total lesion glycolysis showed excellent agreement (ICC ≥ 0.92) between manual and SAC Bayesian segmentation methods. The SAC Bayesian classifier is more reproducible and produces similar lesion features compared to manual segmentation, giving the best concordant results of all other methods. Deep learning–based segmentation can achieve overall good segmentation results but failed in few patients impacting patients’ clinical evaluation.

**Supplementary Information:**

The online version contains supplementary material available at 10.1007/s10278-023-00823-y.

## Introduction

Lymphomas are one of the most heterogeneous groups of cancers having a different biological behavior depending on the type and degree of differentiation. Its diagnosis, staging, and therapy response assessment are nowadays evaluated using [^18^F]FDG PET/CT imaging for the majority of lymphoma types [1, 2]. As for other oncological diseases [3–5], quantification based on maximum standardized uptake value (SUV_max_), mean standardized uptake value (SUV_mean_), metabolic tumor volume (MTV), and total lesion glycolysis (TLG) has demonstrated prognostic value and prediction of treatment outcome in various lymphoma subtypes [6–8]. Moreover, the use of features reflecting tumor burden dissemination (spatial measures) has improved the prediction of outcomes in diffuse large B-cell lymphoma (DLBCL) patients [9, 10].

Despite promising results with disease quantification, quantitative evaluation of [^18^F]FDG PET/CT imaging is not performed, as often as it should be, due to the need for careful lesion segmentation and further feature extraction. In addition, standardized and harmonized image acquisition and reconstruction protocols are mandatory. Manual segmentation is a challenging and time-consuming task, especially for lymphoma patients with a heavy disease burden. Besides, manual segmentation is susceptible to natural intra- and inter-observer variability. Nevertheless, manual segmentation by an expert is considered the segmentation ground truth. Reproducible and accurate semiautomatic and/or fully automatic segmentation methods are of paramount importance to provide comprehensive lymphoma disease quantification using a PET-directed personalized approach for lymphoma patients.

For clinical and research use, several PET tumor segmentation tools have been purposed and validated. However, beneficial evidence remains more frequently reported for the lung, and head and neck tumors [11]. For lymphoma [^18^F]FDG-avid lesions, segmentation presents additional challenges since the disease may be spread throughout the entire body, with several distant localizations, large variability in appearance, and sometimes difficult to distinguish from normal high uptake regions. Methodologies for lymphoma [^18^F]FDG PET segmentation have been proposed, ranging from thresholding to deep learning, but there is no consensus on how the entire disease volume should be segmented. Several studies using quantification of [^18^F]FDG-avid lymphoma lesions applied fixed or adaptive threshold techniques [7]. Recently, efforts have been made to develop a fully automatic segmentation method using deep learning specifically for DLBCL [12]. Despite promising results, verification of all lesions detected and segmented is still needed for clinical use and method validation in large independent datasets is essential. Furthermore, the ultimate purpose of segmentation is the quantification of the disease. Few studies have reported the effect of the different segmentation methods on the lesion’s features outcome [13].

This study has three main goals: (a) to validate improved versions of *k*-means and Bayesian clustering segmentation methods on [^18^F]FDG-avid lymphoma lesions; (b) to compare these improved methods against their standard versions, threshold-based methods, manual segmentation, and also against a semiautomatic and two fully automatic deep learning–based segmentation approaches based on a state-of-the-art deep learning framework; (c) to assess the influence of these segmentation methods on lesion characterization, using features based on uptake, geometry, and dissemination.

## Materials and Methods

### Patient Population

This retrospective and single-center study included 65 patients naive to treatment randomly selected (mean age: 64 years; age range: 24–95 years; mean body mass index of 25 kg/m^2^ and ranging from 17 to 37 kg/m^2^; 37 women) who were diagnosed with B-cell lymphoma from 2009 to 2018 (16 Hodgkin lymphoma; 29 follicular non-Hodgkin lymphoma, 19 DLBCL, and 1 low-grade non-Hodgkin lymphoma). For each patient, only the disease staging [^18^F]FDG PET/CT image was included (6 stage I, 9 stage II, 17 stage III, and 33 stage IV). The study was approved by the Ethics Committee of the Champalimaud Foundation.

### Image Acquisition

All patients were scanned in the Philips Gemini TF 16 PET/CT after intravenous injection of 3.5 ± 0.20 MBq/kg. Image reconstruction was performed onsite with manufacturer standard clinical parameters (Table 1 of the Supplementary Material). The scanner and reconstruction protocol is in accordance with the European Association of Nuclear Medicine (EANM) Research GmbH standard 1 (EARL1) with regular calibration and quality control according to the EANM guidelines [14]. The images’ SUV calculation was based on the injected activity and body weight.

### Lesions Segmentation

**Fig. 1:**
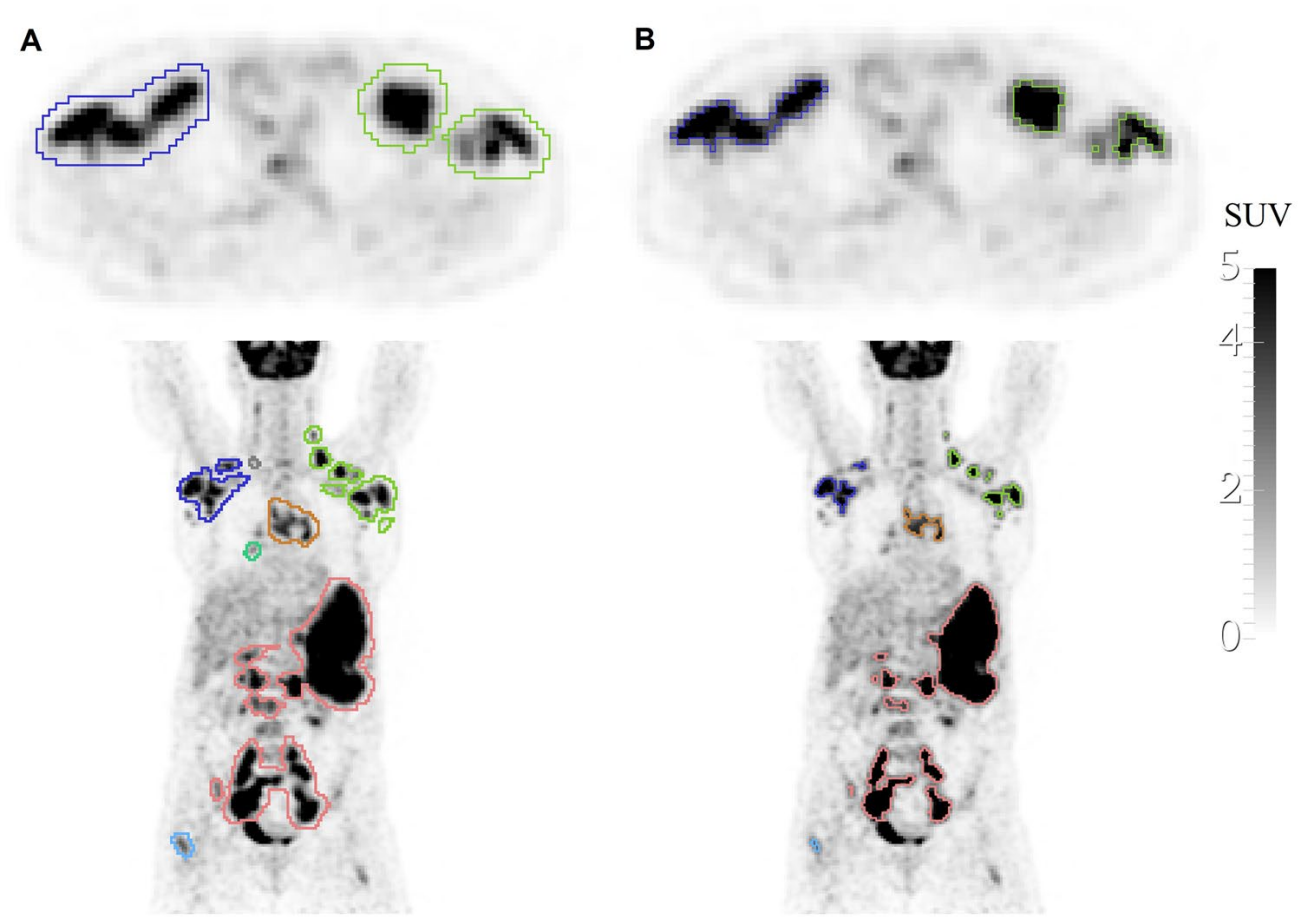
An example of the ROI definition (initialization for the semiautomatic methods) in a PET image of a lymphoma patient **A** and the corresponding segmentation result using a semiautomatic method (SAC Bayesian). **B** Different colors represent different disconnected lesions

All visible lymphoma lesions or clusters of lesions, as stated in the nuclear medicine reports, including splenic and liver diffuse involvement, were identified by experienced nuclear medicine physicians on the more convenient image plane (in most cases, the axial plane). This task was divided among six nuclear medicine physicians (five physicians with more than 7 years and less than 13 years of experience and one with more than 25 years of experience with [^18^F]FDG PET/CT). Then, two experienced observers, Obs1 and Obs2 (both nuclear medicine researchers, one with 4 years of experience and the other with 10 years of experience, respectively), drew a large 3D region of interest (ROI) around each lesion, ensuring that the whole lesion and surrounding background tissue were within the ROI, but without including nearby normal high uptake tissue (see an example in Fig. 1A). Physicians and observers could adjust the level of fusion between the PET and CT to facilitate the identification of the lesion. They could also use gray or other color scales. These ROIs were then used, as an initialization, to feed the classical semiautomatic segmentation algorithms (see below). The same two observers also performed manual segmentation on all identified lesions in a slice-per-slice manner. ROI definition and manual segmentation were performed in 3D Slicer 4.11.2 software (https://www.slicer.org). The time consumed on each one of the previous tasks (ROI definition and manual segmentation) was registered for both observers.

### Classical Semiautomatic Segmentation

In addition to manual segmentation, we implemented and tested different automatic segmentation methods inside of the 3D ROI previously drawn, which we call semiautomatic because they need an initialization (3D ROI). These methods are based on thresholding and clustering. The algorithms were implemented in C +  + language using the Insight Segmentation and Registration Toolkit (ITK) (www.itk.org) and CImg (http://cimg.eu/) software libraries. An open-source implementation of these semiautomatic segmentation algorithms (Microsoft Windows version) is available upon request and can be integrated as an extension into 3D Slicer software platform version 4.11.2 or higher, for an integrative and user-friendly application. The thresholding and clustering methods implemented are briefly described below.

Two threshold-based methods were applied in this study: an absolute threshold of SUV (SUV of 2.5) and a relative one using a percentage of the maximum SUV in each lesion (41% of SUV_max_). The SUV threshold of 2.5 has been reported as optimal to measure baseline MTV in DLBCL, giving the best inter-observer agreement among other threshold methodologies [15], and has been generally chosen in lymphoma studies [16, 17]. A threshold of 41% of the SUV_max_ within the lesion was also applied, as it is recommended by the EANM for solid tumors [18], and has been applied in patients with Hodgkin lymphoma [7, 19], DLBCL [7, 20], and peripheral T-cell lymphoma [21].

Two standard clustering segmentation methods were also implemented: *k*-means and Bayesian (both with 2 classes). *k*-means is frequently used due to its simplicity [22]. It iteratively assigns each voxel’s intensity to the nearest cluster centroid. In this case, the mean SUV of the cluster is considered the centroid. The initialization for the centroids (*k*-initial cluster centers) was calculated based on the percentiles of the SUV distribution within the ROI delineated. Then, the algorithm uses a *k*-means statistical classifier to iteratively attribute each voxel into a cluster and update the centroids. Bayesian clustering algorithms allow noise statistical modeling; thus, they are less sensitive to noise than other classifiers [23]. This unsupervised classifier estimates the probability of a given voxel belonging to a cluster. The decision on the voxel class is based on the maximum a posteriori likelihood. In the present implementation, the SUV of each class is modeled using a Gaussian mixture model.

For clustering methods, only two classes/clusters need to be defined for segmentation (lesion and background). However, in some situations, this solution includes too many or too few voxels in the lesions, compared to manual segmentation. This can be minimized using, for instance, 3 classes and then merging two of them (the two highest or the two lowest) (see Fig. 1 of Supplementary Materials). The problem here is to automatically define the number of classes to be initially considered and, if necessary, the classes to be merged. We have developed a self-adaptive configuration (SAC) solution that can be used in the *k*-means and Bayesian clustering methods. It is contrast-orientated and depends on the lesion-to-background values [24].

#### Self-Adaptive Configuration Optimization

The algorithm starts by diving the sample (voxels’ intensities) automatically into 3 classes, according to the segmentation process chosen (*k*-means or Bayesian). Thereafter, a coefficient (Eq. (1)) is calculated, and then, depending on their value, the classes to merge will be the two with lowest intensities or the two with highest intensities, or the program runs again the segmentation method but now defining only 2 initial classes. The coefficient is calculated by,

1 $$coef=({m}_{3}-{m}_{1})/{(m}_{3}+{m}_{1})$$

where ${m}_{3}$ is the mean SUV of the voxels in class 3 (with higher values) and ${m}_{1}$ the mean SUV of the voxels in the class with lower values (class 1). This coefficient is similar to the asymmetry index frequently used in other contexts.

The empirical rule created based on prior knowledge defines the number of classes to be created and the criterion for the merging (if necessary). The rule is defined as follows, depending on the coefficient ($coef\in \left[\mathrm{0,1}\right]$),

2 $$\left\{\begin{array}{c}\mathrm{if}\;coef <0.90, \text{merge the two lower classes}\\ \\ \text{if coef}\;\ge\;\text{0.94, merge the two higher classes}\\ \\ \text{otherwise, do the segmentation with just 2 classes}\end{array}\right.$$

In practice, this rule means that when the voxels’ intensities are divided into three classes (“background,” “border,” and “lesion”) if there is a “big” relative difference between the “lesion” mean intensity and the “background” mean intensity, it is better to include the “lesion border” inside the “lesion.” If the relative difference is “small,” it is better to include the “lesion border” inside the “background.” Otherwise, it is better to do a simple two classes classification. The optimal thresholds were established and validated in previous work using a different dataset of whole-body [^18^F]FDG PET images from patients with different primary tumors [24].

### Deep Learning–Based Segmentation

Three different approaches were used for segmentation using deep learning–based techniques: (1) a fully automatic method based on a 3D U-Net trained by Blanc-Durand et al. [12]; (2) a fully automatic method based on a 3D U-Net trained by us; (3) a semiautomatic approach based on a 3D U-Net trained by us. All these three networks were built and trained using the nnU-Net, a state-of-the-art framework for biomedical image segmentation based on a 3D full-resolution U-Net proposed by Isensee et al. [28]. This framework estimates the optimal network architecture and hyperparameters based on heuristic rules. For the three approaches, a standard U-Net architecture composed of an encoder and a decoder network with skipped connections between the two paths was applied. The networks were trained using fivefold cross-validation with a random split into 80% for training and 20% for internal validation.

#### Fully Automatic Deep Learning–Based Segmentation

The first fully automatic deep learning–based segmentation here used is a trained 3D U-Net publicly available. It was trained by Blanc-Durand et al. [12] using a dataset of 639 DLBCL patients and two input channels: PET and CT. It achieved a median validation Dice coefficient (DC) of 0.79 and interquartile range (IQR) of [0.66 to 0.87] in the internal validation performed by the authors.

The second fully automatic deep learning–based segmentation is also a 3D U-Net, but trained by us using a dataset of 144 whole-body [^18^F]FDG PET images from lymphoma patients (46 ± 19 years old, 68 women) extracted from The Cancer Imaging Archive [25] published by the autoPET MICCAI challenge [26, 27]. All images were acquired in a single PET/CT scanner (Siemens Biograph mCT), and all FDG-avid lymphoma lesions were manually segmented by a radiologist and a nuclear medicine physician in consensus. This 3D U-Net was trained on an Ubuntu 20.04 Windows Subsystem for Linux (WSL) in a computer equipped with a NIVIDA RTX A6000 graphics.

#### Semiautomatic Deep Learning–Based Segmentation

In this approach, a 3D U-Net was trained on small patches of 64 × 64 × 64 voxels which correspond to 256 × 256 × 256 mm^3^. The same dataset as previously described from the autoPET MICCAI challenge was used. In total, 2537 isolated lesions were identified and used to extract the patches centered on them. This simulates a real-world application, where the physician should indicate approximately the center of the lesion he/she wants to segment to start the deep learning–based segmentation. The 3D U-Net was trained in the same computer previously indicated.

For testing the trained network in our dataset, for each lesion identified by the physician, based on ROI delineation previously described, a patch centered on it was extracted and given as input to the network for lesion segmentation. Thus, only patches containing lymphoma lesions were given for segmentation. All other regions not contained in the patches were considered not having lymphoma. This network could be used for fully automated segmentation but was not optimized for it. Thus, it was here tested only in patches containing lesions.

### Lesion Quantification

In this work, the goal of lesion quantification is to evaluate the influence of different segmentation methods on potential clinically relevant lymphoma lesion features. Quantitative lesion features were extracted automatically after patients’ lesions segmentation. Thirty-one features based on intensity (SUV scale), geometry, and spatial distribution were computed. Implementation of intensity and geometric features were based on the definitions given by Aerts et al. [29], except for SUV_peak_ and TLG. SUV_peak_ was measured in neighborhoods of 3 × 3 × 3 voxels. TLG was obtained by multiplying MTV by the SUV_mean_. Moreover, as tumor burden dissemination measures have shown prognostic value [9], 8 dissemination features were also implemented and calculated. These are described in detail in Supplementary Material.

### Statistical Analysis

To evaluate the proportion of overlap between segmentation methods, the DC was calculated between manual segmentations and the semi- and fully automatic segmentations. Then, the same coefficient was used to evaluate inter-observer manual segmentation; intra-observer manual versus semiautomatic segmentation; and inter-observer semiautomatic segmentation. For statistical inference, Friedman and Wilcoxon tests were used with a significance level of 5%.

Agreement between lesion features extracted from manual and semi- and fully automatic segmentations was measured using intraclass correlation coefficient (ICC) for absolute agreement. The agreement was computed on a patient basis, i.e., considering all patients’ tumor burden, and also on a representative lesion basis, as has been recommended to characterize lymphoma [30, 31].

Statistical analysis was performed using the IBM SPSS version 26 and R software (version 3.2.5, https://www.r-project.org/).

## Results

**Fig. 2:**
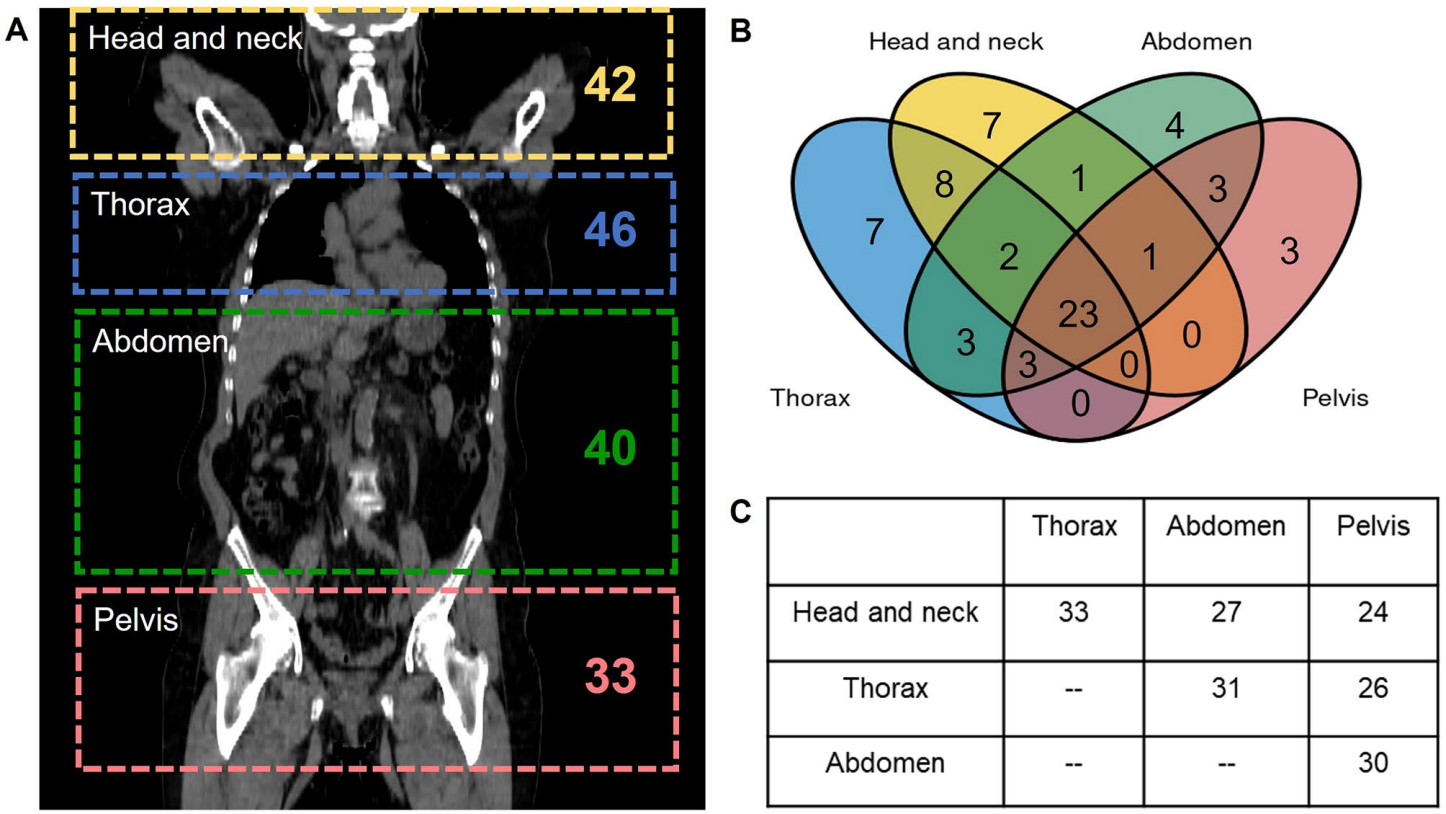
**A** Number of patients with lesions located in the head and neck, thorax, abdomen, and/or pelvis. **B** Venn diagram representing the distribution of lesions location in lymphoma patients included in the dataset. **C** The number of patients with lesions located at least in two different anatomical locations

**Fig. 3:**
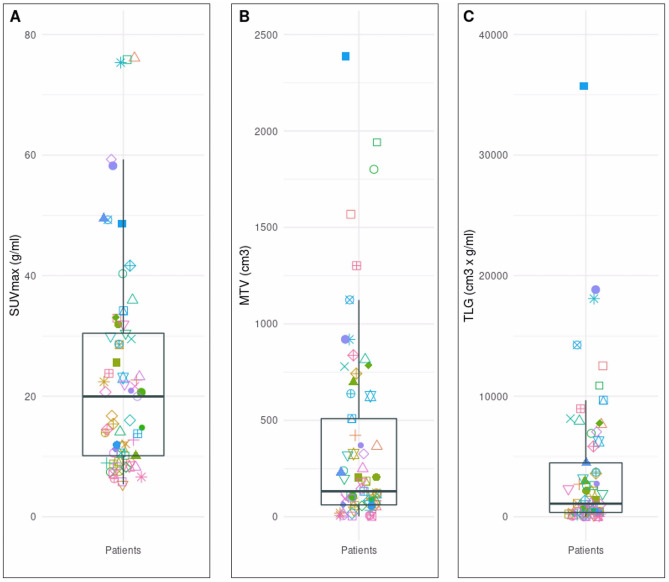
Patients’ tumor burden characterization using the mean SUV_max_ **A**, mean MTV **B**, and mean TLG **C** obtained from the two manual segmentations (mean value of the features from both segmentations were used). Each combination of symbol and color represents a patient

A total of 920 lymphoma lesions (single and/or clusters) were identified in the [^18^F]FDG PET/CT images from the 65 patients (1st, 2nd, and 3rd quartile of lesions’ SUV_max_ and MTV, respectively: 4.49, 6.20, and 10.32 and 1.34 cm^3^, 3.01 cm^3^, and 9.66 cm^3^). The anatomical localization of the patient’s lesions is represented in Fig. 2. For patients’ total tumor burden characterization, the mean of SUV_max_, MTV, and TLG obtained from manual segmentations of both observers is presented in Fig. 3.

### Segmentation Assessment

**Fig. 4:**
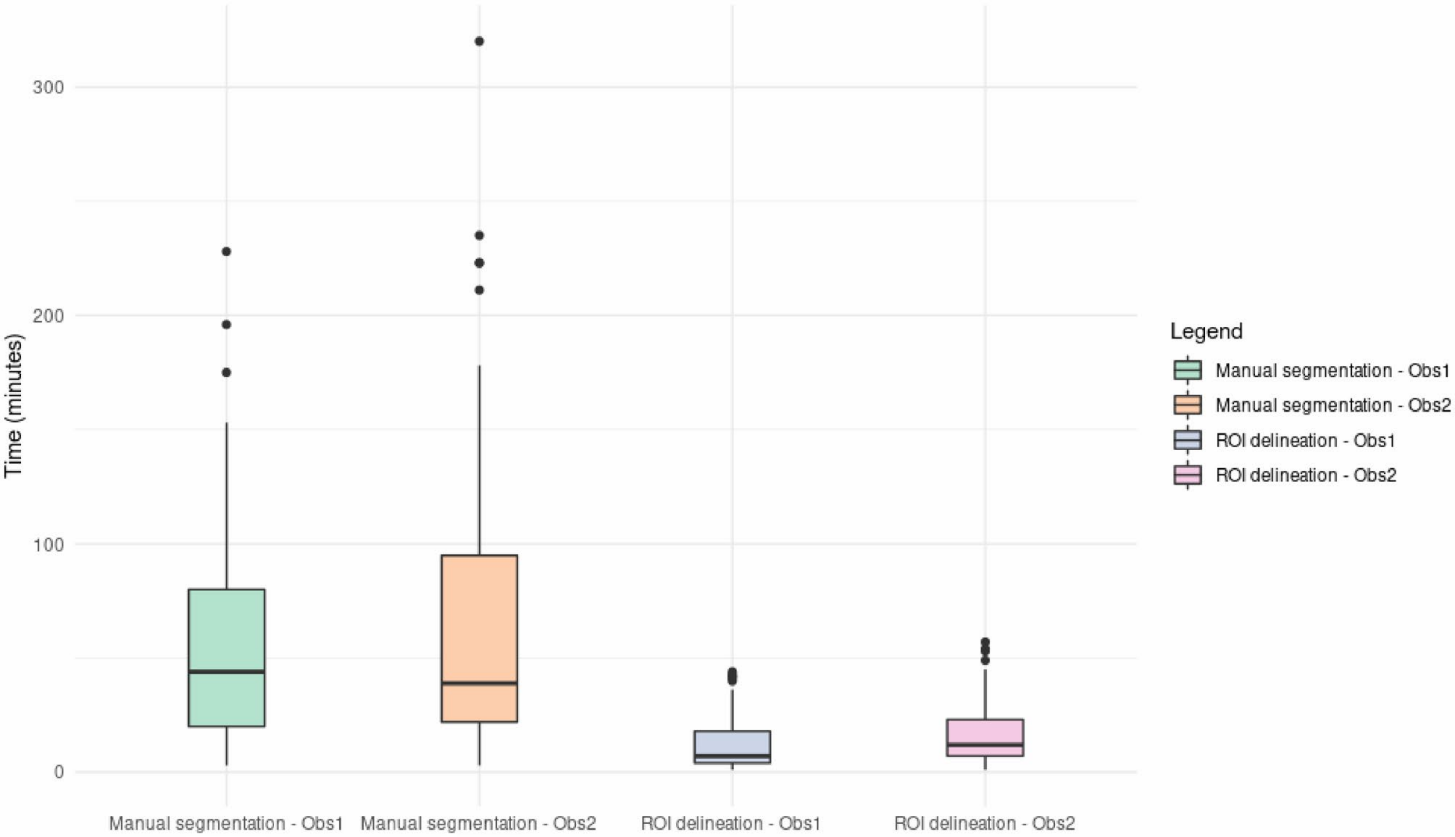
Distribution of the time consumed in manual segmentation and ROI delineation for each [^18^F]FDG PET image by two experienced observers (Obs1 and Obs2)

Figure 4 illustrates the distribution of time consumed by observer per patient. The classical semiautomatic segmentation methods used were approximately five times faster than the manual one (*p* < 0.001, Wilcoxon test): median time of 8 min (range 1 to 57 min) versus 42 min (range 3 to 320 min). The time needed for the classical semiautomatic segmentation was almost exclusive for the manual delineation of the 3D ROI. The automatic part is negligible (less than a second in most patients). The time needed by the physician to identify the lesions was not measured but may take several minutes depending on the number and localization of the lesions.

**Fig. 5:**
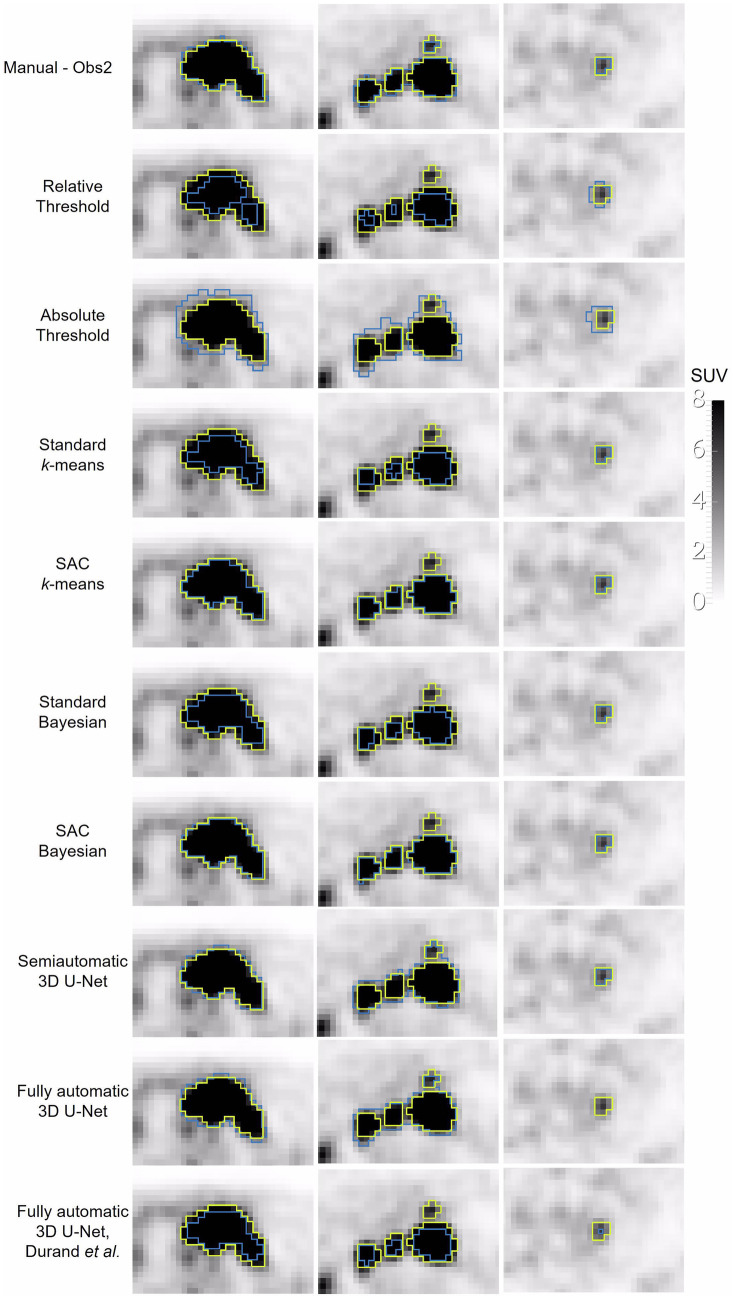
Examples of segmentation results in three independent lymphoma lesions using semiautomatic, fully automatic, and manual segmentation from the observer 2 (Obs2), in blue, in comparison with manual segmentation of observer 1, in yellow

**Fig. 6:**
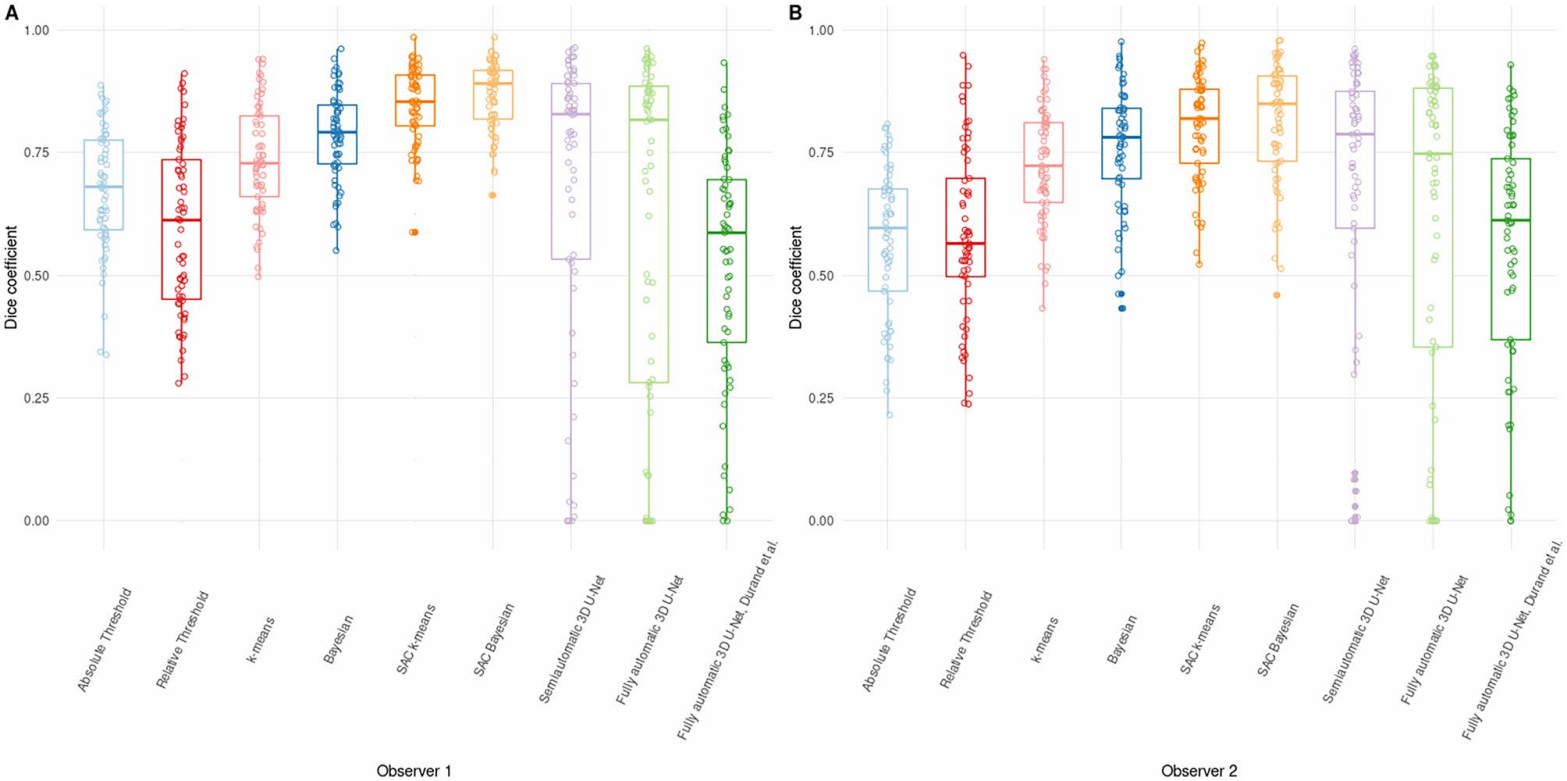
Box plots of the Dice coefficients between manual segmentation (two observers) and the seven semiautomatic and two fully automatic segmentation methods. Results **A** considering manual segmentation of observer 1 as reference, and **B** considering manual segmentation of observer 2 as reference (*SAC*, self-adaptive configuration)

Regarding the assessment of the semiautomatic and fully automatic segmentations, an example of the resulting segmentation for 3 lesions is shown in Fig. 5. The DC distribution obtained from the semiautomatic and fully automatic segmentations having the manual segmentations as ground truth is represented in Fig. 6. Values were computed on a patient basis (total tumor burden). The lowest median DC was achieved with the fully automatic model published by Blanc-Durand et al. and threshold-based segmentation methods (median DC ranging from 0.56 to 0.68). SAC Bayesian method achieved the best overlap with manual segmentation: median DC of 0.89 and 0.85 for Obs1 and Obs2, respectively. These values are slightly superior to DC obtained between both manual segmentations (median DC = 0.84), however not statistically different (Friedman test, *p* = 0.148). Table 1 shows the DC obtained using the SAC Bayesian method. The median inter-observer DC achieved between SAC Bayesian was significantly higher (Wilcoxon test, *p* < 0.001) than between inter-observer manual segmentation (DC = 0.94 versus DC = 0.84). Comparatively to the inter-observer manual segmentation, higher median DC was constantly accomplished between manual and semiautomatic SAC Bayesian segmentation, both intra- and inter-observer. Results for the other semiautomatic and fully automatic methods are in Table 2 of Supplementary Material. There were no statistically significant differences between the DC achieved among different subtypes of lymphoma (*p*-value ≥ 0.36, Kruskal–Wallis test), independently of the observer or segmentation method.

**Table 1:** Dice coefficient between manual and SAC Bayesian (SAC-B) segmentation methods for both observers (Obs1 and Obs2)

Segmentations	Median	Interquartile Range	Maximum	Minimum
Manual (Obs1) vs manual (Obs2)	0.84	0.14	0.97	0.45
SAC-B (Obs1) vs SAC-B (Obs2)	0.94	0.07	1.00	0.61
Manual (Obs1) vs SAC-B (Obs1)	0.89	0.10	0.98	0.66
Manual (Obs1) vs SAC-B (Obs2)	0.87	0.13	0.97	0.52
Manual (Obs2) vs SAC-B (Obs2)	0.85	0.18	0.98	0.47
Manual (Obs2) vs SAC-B (Obs1)	0.86	0.16	0.97	0.46

In terms of patient-based analysis, semiautomatic deep learning, fully automatic deep learning, and fully automatic by Blanc-Durand et al. presented lower DC compared with classical semiautomatic methods in 9, 11, and 8 patients, respectively (DC < 0.22). These poorer DC were found with all three approaches in the same 7 patients of these cohorts. No pattern of lesions localization and uptake was found in these 7 patients. This because the lesions close to normal high uptake regions in the whole body (brain, liver, kidneys, and bladder) were not identified and segmented. Furthermore, the correct identification and segmentation of abdominal lesions was difficult. For the 3D U-Net of Blanc-Durand et al. that was trained with DLBCL patients, we highlight that these 8 cases were from patients diagnosed with DLBCL (*N* = 3) and FL (*N* = 5). No patterns for the SUV_max_ or MTV were found in these patients with the lowest DC. Nevertheless, semiautomatic and fully automatic deep learning–based methods trained with the dataset from autoPET MICCAI challenge had a median DC very close to SAC-based methodologies and to the median DC obtained from both observers’ manual segmentation. Of note, both deep learning–based segmentation methods trained with the autoPET MICCAI challenge dataset originated statistically higher DC than the 3D U-Net of Blanc-Durand et al. (*p* < 0.05, Wilcoxon test).

### Lesion Features Reproducibility

Regarding the three deep learning–based segmentation methods (semi- and fully automatic), we found out that in 20 out of 65 patients, the lesions SUV_max_ was wrong. Interestingly, in some of these patients, the lesion with the highest SUV_max_, based on the physician identification, was correctly identified but non-malignant regions with higher SUV_max_ were also identified and segmented. For these reasons, we decided not to report any more quantitative analysis on the “lesions” segmented using the deep learning–based segmentations.

On a patient basis analysis, the SAC Bayesian method produced the best lesion features reproducibility, having the features from manual segmentation as the reference. An excellent agreement (ICC ≥ 0.92) was obtained for the frequently used features SUV_max_, SUV_mean_, SUV_peak_, MTV, and TLG (Table 2). The results for the remaining features are in Table 3 of Supplementary Material. The agreement was higher or equal between the two semiautomatic segmentations obtained from the SAC Bayesian method than between the two manual segmentations, for all features. Note that all features related to the disease dispersion were very reproducible (ICC ≥ 0.97), both for the manual and semiautomatic SAC Bayesian segmentation methods.

**Table 2:** ICC between features from manual and SAC Bayesian (SAC-B) segmentations on a patient basis (total tumor burden) and on a single lesion basis (patient’s lesion with highest TLG and highest SUVmax), for both observers (Obs1 and Obs2)

**Features**	**Segmentations**					
	**Intra-observer**		**Inter-observer**			
	**Manual (Obs1)** **vs** **SAC-B (Obs1)**	**Manual (Obs2) vs** **SAC-B (Obs2)**	**Manual (Obs1) vs** **SAC-B (Obs2)**	**Manual (Obs2) vs** **SAC-B (Obs1)**	**SAC-B (Obs1) vs** **SAC-B (Obs2)**	**Manual (Obs1)** **vs** **Manual (Obs2)**
Total tumor burden						
SUV_max_	1.00	1.00	1.00	1.00	1.00	**1.00**
SUV_peak_	1.00	1.00	1.00	1.00	1.00	**1.00**
SUV_mean_	0.94	0.97	0.95	0.95	1.00	**0.98**
MTV	0.96	0.92	0.94	0.96	0.99	**0.97**
TLG	0.99	0.99	0.99	1.00	1.00	**1.00**
Based on the lesion with the highest TLG						
SUV_max_	1.00	1.00	1.00	1.00	1.00	**1.00**
SUV_peak_	1.00	1.00	1.00	1.00	1.00	**1.00**
SUV_mean_	0.95	0.96	0.96	0.94	1.00	**0.99**
MTV	0.96	0.90	0.95	0.90	0.98	**0.94**
TLG	0.98	0.98	0.99	0.97	0.99	**0.99**
Based on the lesion with the highest SUV_max_						
SUV_max_	1.00	1.00	1.00	1.00	1.00	**1.00**
SUV_peak_	1.00	1.00	1.00	1.00	1.00	**1.00**
SUV_mean_	0.90	0.94	0.91	0.92	0.99	**0.97**
MTV	0.85	0.60	0.70	0.68	0.81	**0.86**
TLG	0.76	0.72	0.76	0.72	0.94	**0.97**

Statistical good agreement was also obtained from most of the features obtained from the *k*-means, SAC *k*-means, and Bayesian segmentations (Supplementary Material Tables 4, 5, and 6), however, in general, slightly inferiors to the ones obtained from the SAC Bayesian segmentation. Lesion features from the threshold-based segmentation methods originated the overall worst agreement with the features obtained from the manual segmentations (Supplementary Material Tables 7 and 8).

The ICC between the SUV_max_, SUV_mean_, SUV_peak_, MTV, and TLG extracted from the manual and SAC Bayesian segmentation, considering just the highest TLG lesion and highest SUV_max_ lesion as the disease’s representative, is also in Table 2. Excellent ICCs were achieved (ICC ≥ 0.94) considering the highest TLG lesion. Considering the highest SUV_max_ lesion, the ICC is also very high (ICC ≥ 0.90) for the SUV_max_, SUV_peak_, and SUV_mean_, but inferior for MTV and TLG. There were statistically significant differences between the features calculated based on the highest TLG lesion and highest SUV_max_ lesion as the disease’s representative (*p* < 0.01, Wilcoxon test). In some cases, the lesion with the highest SUV_max_ was within a large conglomerated, and in one segmentation method was connected to its neighbors, and in other segmentations was disconnected from its neighbors. In practice, the segmentation difference is minor, differentiating in just a few voxels, and thus has an insignificant impact when the disease is characterized by the total tumor burden.

## Discussion

This is the first study addressing a quantitative comparison, based on 31 lesions’ features, between manual, semi-, and fully automatic segmentation methods on a set of whole-body [^18^F]FDG PET/CT images from patients with lymphoma. The number of patients and lesions/clusters is considerably higher than what other studies [19, 32, 33] have reported within the clinical setting of lymphoma.

Threshold-based methods and publicly available 3D U-Net from Blanc-Durand et al. demonstrated weaknesses (inferior median DC) in comparison with clustering-based methods and semi- and fully 3D U-Net trained in this work. Clustering-based methods showed a very good agreement with manual segmentations, especially the SAC version of the Bayesian classifier. The results from inter-observer variability showed that using the SAC Bayesian method, the DC (segmentation overlap) was higher between the SAC Bayesian segmentation (both observers) than between the two manual segmentations, therefore suggesting that this semiautomatic segmentation method may be a better option than the simple manual segmentation. Good results were also obtained from the SAC *k*-means. Threshold-based methods depend on the SUV value that is strongly influenced by the physical (real) image resolution of the scanner, especially the SUV_max_. This reinforces the inadequacy of using fixed threshold methods, especially if no harmonized reconstruction protocols are used among different scanners.

A state-of-the-art deep-learning framework [28] was applied for training deep learning–based segmentation, both for semi- and fully automatic. For the network published by Blanc-Durand et al., one of the top-rated deep learning networks published and available in the literature, the median DC obtained was comparable with the threshold-based methods. This decrease in network performance in an external test set may be due to the ground truth used for the training process. Although Blanc-Durand et al. trained the network on a large dataset (639 whole-body images), a relative threshold (41% of SUV_max_) was used to generate the ground truth for training. Thus, it may have compromised the results when this network is compared with manual segmentation.

Both 3D U-Net trained in this work for semi- and fully automatic segmentation showed promising results. A good agreement with manual segmentation was achieved (Supplementary Table 2), very close to SAC-based clustering methods. This may be due to the ground truth used for training being the manual segmentation. However, in a patient basis analysis, the results were inferior to SAC-based approaches since the segmentations originated incorrect patient’s total tumor burden SUV_max_ in 20 out of 65 patients. This is unacceptable for clinical practice usage autonomously.

As shown in our study, there are still several difficulties when using deep learning–based approaches. External validation with independent datasets usually leads to a decrease in the performance of the models [34]. Clinical human supervision is still indispensable, and in some patients, the time needed for the verification and adjustment of the segmentation may be close to the time needed for the classical semiautomatic or manual segmentations. The construction of large and accurate datasets (of sparse existence) needed to train, with reliable manual or semiautomatic segmentations, is a demanding and time-consuming task. Nevertheless, we believe that the future will pass through fully automatic segmentation methods, using deep learning or other technology. The reproducible SAC Bayesian method may be an excellent option to build accurate large datasets to train/validate deep learning models.

For lesion characterization, the features extracted from the SAC Bayesian segmentation are very close to the ones extracted from the manual segmentation for all patients. The SAC *k*-means, *k*-means, and Bayesian segmentation methods originated an overall good agreement between the features extracted in comparison with manual segmentation. However, their agreement with manual segmentation features is, in general, inferior to the obtained with SAC Bayesian. This reinforces the adequacy of this semiautomatic segmentation method.

More often than not, lymphoma patients present with multiple lesions, rendering the segmentation methodology a very tough and challenging activity. Thus, some studies [30, 31] have recommended the use of a single representative lesion to characterize the disease. We used two criteria to define the representative lesion: the one with the highest SUV_max_ and the one with the highest TLG. The results also showed an excellent agreement (ICC ≥ 0.90 for SUV_max_, SUV_peak_, SUV_mean_, MTV, and TLG) when the lesion used was the one with the highest TLG. Poorer results were obtained when considering the lesion with the highest SUV_max_ for features that depend on the lesion volume (MTV and TLG). This suggests that using only the lesion with the highest SUV_max_ is not as reproducible as using the lesion with highest TLG or all tumor burden. A further study designed to evaluate the expected added value of using total tumor burden instead of the lesion with highest TLG is ongoing.

In the present work, besides the reproducibility of the traditional first-order and geometric lesion features, the reproducibility of 8 features representing the tumor/disease spreading was also assessed. As expected, the results showed excellent reproducibility both for manual and classical semiautomatic methods, independently of the technique used (ICC ≥ 0.93 for all 8 features).

There are several limitations to our study. First, our dataset comprises just 65 lymphoma patients, including 920 lesions for analysis. Larger numbers of lymphoma patients than the ones used to validate the several different methods may improve the results herein described. Another limitation is the use of external datasets (different scanners, reconstruction protocols, and NM physicians identifying and/or segmenting the lesions) to train the deep learning–based techniques. This renders the performance of the networks when applied to our independent dataset an even more difficult task. In addition, our single-scanner and single-center study may limit the reproducibility of our results, despite using EARL1 harmonized guidelines. Assessment of physicians’ agreement in the identification of the lesions was not part of this work but is highly important. It would make the comparison of semiautomatic and fully automatic segmentation methods fairer than leaving it out.

## Conclusion

The proposed semiautomatic segmentation method based on a SAC Bayesian classifier is very robust and more reproducible than manual segmentation. In addition, it is faster than manual segmentation, producing similar lesion features. This method can replace manual segmentation of lymphoma lesions contributing to more consistent quantitative measures with potential to build accurate large datasets to train/validate deep learning–based segmentation and improve models’ performance.

Absolute or relative threshold-based segmentation methods should be avoided, especially in clinical studies, since important lesion features such as SUV_mean_, MTV, and TLG may be substantially different from the ones obtained with manual segmentation.

Deep learning–based segmentation methods showed promising results but are still not robust enough to be applied autonomously for lymphoma lesions segmentation in clinical practice.

## Supplementary Information

Below is the link to the electronic supplementary material.

Supplementary file1 (DOCX 167 KB)

## Data Availability

An open-source implementation of the semiautomatic segmentation algorithms (Microsoft Windows version) is available upon request and can be integrated as an extension into 3D Slicer software platform version 4.11.2 or higher, for an integrative and user-friendly application.

## Abbreviations

SUV: Standardized uptake value
MTV: Metabolic tumor volume
TLG: Total lesion glycolysis
DLBCL: Diffuse large B-cell lymphoma
EANM: European Association of Nuclear Medicine
ROI: Region of interest
SAC: Self-adaptive configuration
DC: Dice coefficient
ICC: Intraclass correlation coefficient
