# Supplementary material for: Evaluation of Semiautomatic and Deep Learning–Based Fully Automatic Segmentation Methods on [18F]FDG PET/CT Images from Patients with Lymphoma: Influence on Tumor Characterization
Source: J Digit Imaging. 2023 Apr 14;36(4):1864–76. doi: 10.1007/s10278-023-00823-y (PMC10407010; doi:10.1007/s10278-023-00823-y)
Supplement: Supplementary file 1 — Supplementary file1 (DOCX 167 KB) [file 10278_2023_823_MOESM1_ESM.docx]

**Supplementary Material**

**Description of the dissemination features calculated:**

In addition to intensity and geometry-based features, 8 dissemination features were implemented and calculated: mean and maximum distance between all lesions’ voxels (MeanDist and MaxDist); mean and maximum distance between the lesions’ SUVmax voxel (MeanDistSUVmax and MaxDistSUVmax); mean and maximum distance between the lesions’ geometric centroid (MeanDistcentroid and MaxDistcentroid) and, finally, the mean and maximum distance between the voxel with maximum SUV in the lesion with higher TLG and the voxels with maximum SUV of the remaining lesions (MeanDistTLGmax and MaxDistTLGmax)

**Region of interest (ROI) division into 3 classes in the segmentation process**


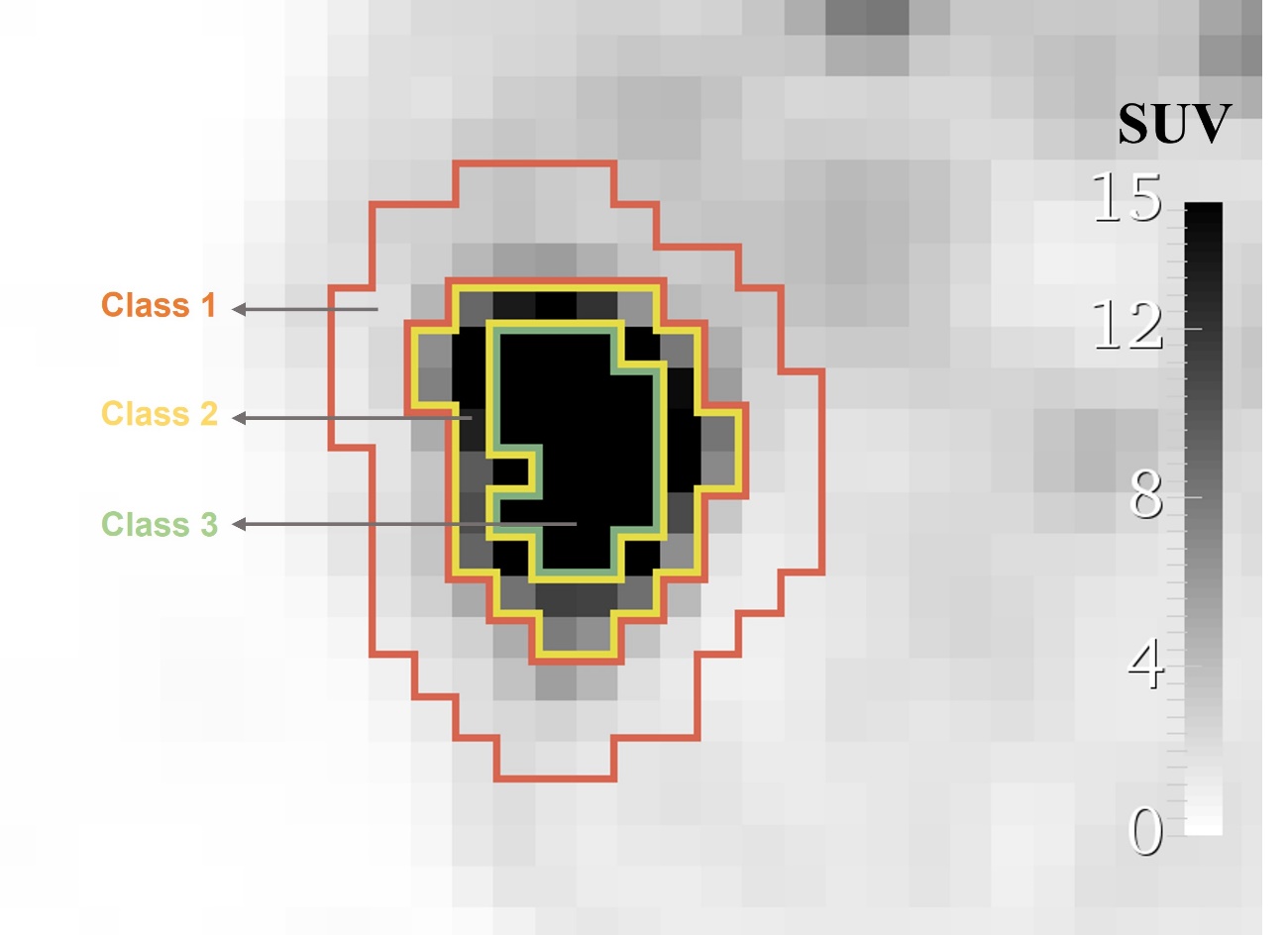
**Fig. 1** Example of a ROI initially divided into 3 classes (from the periphery to the center of the lesion, class 1 is between orange contours, class 2 is between yellow contours, and class 3 is between green contours) in SAC segmentation. The obtained final segmentation was: class 1 – background; and classes 2 and 3 – lesion.

**Manufacturer standard clinical parameters used for image reconstruction:**

**Table 1** Image reconstruction settings.

| Parameter | Standard clinical protocol |
| --- | --- |
| PET | |
| Voxel size | 4$\times$4$\times$4 mm^3^ |
| Reconstruction algorithm | BLOB-OS-TF, 3D |
| Number of iterations × subsets | 3 × 33 |
| Relaxation factor | 0.7 |
| Post-reconstruction filter | None |
| Other parameters for image correction | DECY, RADL, ATT, SCAT, DTIM, RAN, NORM |
| CT | |
| kV | 140 |
| Voxel size | $1.17\times1.17\times5$ mm^3^ |

BLOB-OS-TF = BLOB (rotationally symmetric volume elements) ordered-subsets time of flight; DECY = Decay correction; ATT = attenuation correction; SCAT = scatter correction; DTIM = dead time correction; RAN = randoms correction; NORM = detector normalization.

**Dice coefficient between manual and semiautomatic methods**

**Table 2** Dice coefficient, inter and/or intra-observer, for semiautomatic, fully automatic and manual segmentations for the results achieved with two experienced observers (Obs1 and Obs2). (RT = relative threshold of 41% of SUV_max_; AT = absolute threshold of 2.5; kM = standard *k*-means (2 clusters); SAC-kM = Self-adaptive configuration of k-means; Bayes = standard Bayesian (2 clusters)).

| Segmentations | Median | Interquartile Range | Maximum | Minimum |
| --- | --- | --- | --- | --- |
| Manual (Obs1) vs Manual (Obs2) | 0.84 | 0.14 | 0.97 | 0.45 |
| Relative threshold | | | | |
| Manual (Obs1) vs RT(Obs1) | 0.61 | 0.30 | 0.91 | 0.28 |
| Manual (Obs1) vs RT (Obs2) | 0.59 | 0.28 | 0.91 | 0.28 |
| Manual (Obs2) vs RT (Obs2) | 0.56 | 0.23 | 0.94 | 0.24 |
| Manual (Obs2) vs RT (Obs1) | 0.56 | 0.25 | 0.94 | 0.27 |
| RT (Obs1) vs RT (Obs2) | 0.95 | 0.11 | 1.00 | 0.49 |
| Absolute threshold | | | | |
| Manual (Obs1) vs AT(Obs1) | 0.68 | 0.19 | 0.89 | 0.34 |
| Manual (Obs1) vs AT (Obs2) | 0.66 | 0.22 | 0.86 | 0.28 |
| Manual (Obs2) vs AT (Obs2) | 0.60 | 0.22 | 0.81 | 0.22 |
| Manual (Obs2) vs AT (Obs1) | 0.61 | 0.22 | 0.82 | 0.26 |
| AT (Obs1) vs AT (Obs2) | 0.90 | 0.08 | 0.97 | 0.70 |
| Standard *k*-means | | | | |
| Manual (Obs1) vs kM (Obs1) | 0.73 | 0.17 | 0.94 | 0.49 |
| Manual (Obs1) vs kM (Obs2) | 0.74 | 0.15 | 0.95 | 0.47 |
| Manual (Obs2) vs kM (Obs2) | 0.72 | 0.17 | 0.94 | 0.43 |
| Manual (Obs2) vs kM (Obs1) | 0.73 | 0.17 | 0.95 | 0.38 |
| kM (Obs1) vs kM (Obs2) | 0.93 | 0.09 | 0.99 | 0.71 |
| Self-adaptive configuration of k-means | | | | |
| Manual (Obs1) vs SAC-kM (Obs1) | 0.85 | 0.11 | 0.98 | 0.59 |
| Manual (Obs1) vs SAC-kM (Obs2) | 0.85 | 0.12 | 0.98 | 0.62 |
| Manual (Obs2) vs SAC-kM (Obs2) | 0.82 | 0.16 | 0.97 | 0.52 |
| Manual (Obs2) vs SAC-kM (Obs1) | 0.83 | 0.15 | 0.98 | 0.46 |
| SAC-kM(Obs1) vs SAC-kM (Obs2) | 0.94 | 0.08 | 0.99 | 0.71 |
| Standard Bayesian | | | | |
| Manual (Obs1) vs Bayes (Obs1) | 0.79 | 0.12 | 0.96 | 0.55 |
| Manual (Obs1) vs Bayes (Obs2) | 0.81 | 0.13 | 0.98 | 0.45 |
| Manual (Obs2) vs Bayes (Obs2) | 0.78 | 0.15 | 0.98 | 0.43 |
| Manual (Obs2) vs Bayes (Obs1) | 0.77 | 0.16 | 0.96 | 0.37 |
| Bayes (Obs1) vs Bayes (Obs2) | 0.92 | 0.08 | 0.99 | 0.61 |
| Semiautomatic 3D U-Net | | | | |
| Manual (Obs1) vs 3D U-Net | 0.83 | 0.36 | 0.97 | 0 |
| Manual (Obs2) vs 3D U-Net | 0.79 | 0.30 | 0.96 | 0 |
| Fully automatic 3D U-Net | | | | |
| Manual (Obs1) vs 3D U-Net | 0.82 | 0.60 | 0.96 | 0 |
| Manual (Obs2) vs 3D U-Net | 0.75 | 0.53 | 0.95 | 0 |
| Fully automatic 3D U-Net, Blanc-Durand et al. | | | | |
| Manual (Obs1) vs 3D U-Net | 0.59 | 0.35 | 0.93 | 0 |
| Manual (Obs2) vs 3D U-Net | 0.61 | 0.39 | 0.93 | 0 |

**Intraclass correlation coefficients between lesion’s features extracted from manual and semiautomatic segmentations on a patient basis (total tumor burden), for both observers**

**Table 3** Intraclass correlation coefficients (ICC) between features of patients’ tumor burden extracted from different segmentations: manual and semiautomatic SAC Bayesian (SAC-B).

| Features | **SAC Bayesian segmentation-related agreement** | | | | | |
| --- | --- | --- | --- | --- | --- | --- |
|  | Intra-observer | | Inter-observer | | | |
|  | Manual(Obs1)  vs  SAC-B(Obs1) | Manual(Obs2) vs  SAC-B(Obs2) | Manual(Obs1) vs  SAC-B(Obs2) | Manual(Obs2) vs  SAC-B(Obs1) | SAC-B(Obs1) vs  SAC-B(Obs2) | Manual(Obs1)  vs  Manual(Obs2) |
| SUV_max_ | 1.00 | 1.00 | 1.00 | 1.00 | 1.00 | **1.00** |
| SUV_peak_ | 1.00 | 1.00 | 1.00 | 1.00 | 1.00 | **1.00** |
| SUV_mean_ | 0.94 | 0.97 | 0.95 | 0.95 | 1.00 | **0.98** |
| MTV | 0.96 | 0.92 | 0.96 | 0.94 | 0.99 | **0.97** |
| TLG | 0.99 | 0.99 | 1.00 | 0.99 | 1.00 | **1.00** |
| Energy | 1.00 | 1.00 | 1.00 | 1.00 | 1.00 | **1.00** |
| Entropy | 0.99 | 0.99 | 0.99 | 0.99 | 1.00 | **0.99** |
| Kurtosis | 0.95 | 0.92 | 0.93 | 0.93 | 0.99 | **0.95** |
| Mean absolute Deviation | 0.99 | 1.00 | 1.00 | 1.00 | 1.00 | **1.00** |
| Median | 0.90 | 0.93 | 0.91 | 0.91 | 0.99 | **0.96** |
| Minimum | 0.50 | 0.68 | 0.77 | 0.55 | 0.55 | **0.67** |
| Range | 1.00 | 1.00 | 1.00 | 1.00 | 1.00 | **1.00** |
| Root Mean Square | 0.97 | 0.98 | 0.98 | 0.98 | 1.00 | **0.99** |
| Skewness | 0.93 | 0.92 | 0.92 | 0.91 | 0.98 | **0.92** |
| Standard Deviation | 1.00 | 1.00 | 1.00 | 1.00 | 1.00 | **1.00** |
| Uniformity | 0.97 | 0.96 | 0.97 | 0.97 | 0.99 | **0.95** |
| Variance | 0.99 | 1.00 | 0.99 | 0.99 | 1.00 | **1.00** |
| Coefficient of Variation | 0.89 | 0.90 | 0.90 | 0.89 | 0.98 | **0.94** |
| Surface Area | 0.98 | 0.96 | 0.98 | 0.96 | 0.99 | **0.98** |
| Compactness | 0.96 | 0.95 | 0.96 | 0.95 | 0.99 | **0.97** |
| Spherical disproportion | 0.96 | 0.98 | 0.95 | 0.99 | 0.99 | **0.97** |
| Sphericity | 0.98 | 0.98 | 0.97 | 0.99 | 0.99 | **0.99** |
| Surface to volume ratio | 0.93 | 0.90 | 0.91 | 0.90 | 0.97 | **0.90** |
| MeanDist | 0.99 | 0.99 | 0.99 | 0.99 | 1.00 | **0.99** |
| MaxDist | 1.00 | 1.00 | 1.00 | 1.00 | 1.00 | **1.00** |
| MeanDist_SUVmax_ | 0.99 | 0.98 | 0.98 | 0.99 | 0.99 | **0.99** |
| MaxDist_SUVmax_ | 1.00 | 1.00 | 1.00 | 1.00 | 1.00 | **1.00** |
| MeanDist_centroid_ | 0.99 | 0.98 | 0.98 | 0.99 | 0.99 | **0.99** |
| MaxDist_centroid_ | 1.00 | 1.00 | 1.00 | 1.00 | 1.00 | **1.00** |
| MeanDist_TLGmax_ | 0.97 | 0.98 | 0.98 | 0.98 | 0.99 | **0.97** |
| MaxDist_TLGmax_ | 1.00 | 1.00 | 1.00 | 1.00 | 1.00 | **1.00** |

**Table 4** Intraclass correlation coefficients (ICC) between features of patients’ tumor burden extracted from different segmentations: manual and semiautomatic using standard *k*-means (kM).

| Features | **Standard *k*-means segmentation-related agreement** | | | | | |
| --- | --- | --- | --- | --- | --- | --- |
|  | Intra-observer | | Inter-observer | | | |
|  | Manual(Obs1) vs  kM(Obs1) | Manual(Obs2) vs  kM(Obs2) | Manual(Obs1) vs  kM(Obs2) | Manual(Obs2) vs  kM(Obs1) | kM(Obs1)  vs  kM(Obs2) | Manual(Obs1)  vs  Manual(Obs2) |
| SUV_max_ | 1.00 | 1.00 | 1.00 | 1.00 | 1.00 | **1.00** |
| SUV_peak_ | 1.00 | 1.00 | 1.00 | 1.00 | 1.00 | **1.00** |
| SUV_mean_ | 0.81 | 0.84 | 0.82 | 0.83 | 1.00 | **0.98** |
| MTV | 0.87 | 0.85 | 0.88 | 0.86 | 0.99 | **0.97** |
| TLG | 0.95 | 0.95 | 0.96 | 0.95 | 1.00 | **1.00** |
| Energy | 0.98 | 0.99 | 0.99 | 0.99 | 1.00 | **1.00** |
| Entropy | 0.98 | 0.98 | 0.98 | 0.97 | 0.99 | **0.99** |
| Kurtosis | 0.87 | 0.88 | 0.90 | 0.83 | 0.98 | **0.95** |
| Mean absolute Deviation | 0.95 | 0.96 | 0.95 | 0.94 | 0.99 | **1.00** |
| Median | 0.68 | 0.73 | 0.69 | 0.71 | 0.99 | **0.96** |
| Minimum | 0.25 | 0.47 | 0.45 | 0.31 | 0.64 | **0.67** |
| Range | 0.98 | 0.99 | 0.99 | 0.98 | 0.98 | **1.00** |
| Root Mean Square | 0.89 | 0.91 | 0.89 | 0.90 | 1.00 | **0.99** |
| Skewness | 0.75 | 0.77 | 0.77 | 0.74 | 0.96 | **0.92** |
| Standard Deviation | 0.95 | 0.96 | 0.96 | 0.96 | 1.00 | **1.00** |
| Uniformity | 0.97 | 0.92 | 0.97 | 0.93 | 0.98 | **0.95** |
| Variance | 0.90 | 0.93 | 0.92 | 0.91 | 0.99 | **1.00** |
| Coefficient of Variation | 0.57 | 0.62 | 0.59 | 0.60 | 0.97 | **0.94** |
| Surface Area | 0.92 | 0.92 | 0.93 | 0.92 | 0.99 | **0.98** |
| Compactness | 0.84 | 0.86 | 0.85 | 0.85 | 0.99 | **0.97** |
| Spherical disproportion | 0.92 | 0.95 | 0.93 | 0.94 | 0.99 | **0.97** |
| Sphericity | 0.96 | 0.96 | 0.96 | 0.96 | 0.99 | **0.99** |
| Surface to volume ratio | 0.77 | 0.78 | 0.78 | 0.77 | 0.98 | **0.90** |
| MeanDist | 0.98 | 0.96 | 0.98 | 0.96 | 0.99 | **0.99** |
| MaxDist | 1.00 | 1.00 | 1.00 | 1.00 | 1.00 | **1.00** |
| MeanDist_SUVmax_ | 0.97 | 0.97 | 0.97 | 0.98 | 0.99 | **0.99** |
| MaxDist_SUVmax_ | 0.99 | 0.99 | 0.99 | 0.99 | 0.99 | **1.00** |
| MeanDist_centroid_ | 0.97 | 0.98 | 0.97 | 0.98 | 0.99 | **0.99** |
| MaxDist_centroid_ | 0.99 | 0.99 | 0.99 | 0.99 | 0.99 | **1.00** |
| MeanDist_TLGmax_ | 0.96 | 0.98 | 0.97 | 0.98 | 0.98 | **0.97** |
| MaxDist_TLGmax_ | 0.99 | 1.00 | 0.99 | 0.99 | 1.00 | **1.00** |

**Table 5** Intraclass correlation coefficients (ICC) between features of patients’ tumor burden extracted from different segmentations: manual and semiautomatic using SAC *k*-means (SAC-kM).

| Features | **SAC *k*-means segmentation-related agreement** | | | | | |
| --- | --- | --- | --- | --- | --- | --- |
|  | Intra-observer | | Inter-observer | | | |
|  | Manual(Obs1)  vs  SAC-kM(Obs1) | Manual(Obs2)  vs  SAC-kM(Obs2) | Manual(Obs1)  vs  SAC-kM(Obs2) | Manual(Obs2)  vs  SAC-kM(Obs1) | SAC-kM(Obs1)  vs  SAC-kM(Obs2) | Manual(Obs1)  vs  Manual(Obs2) |
| SUV_max_ | 1.00 | 1.00 | 1.00 | 1.00 | 1.00 | **1.00** |
| SUV_peak_ | 1.00 | 1.00 | 1.00 | 1.00 | 1.00 | **1.00** |
| SUV_mean_ | 0.90 | 0.92 | 0.91 | 0.92 | 0.99 | **0.98** |
| MTV | 0.93 | 0.92 | 0.91 | 0.90 | 0.98 | **0.97** |
| TLG | 0.98 | 0.98 | 0.99 | 0.98 | 1.00 | **1.00** |
| Energy | 1.00 | 0.99 | 0.99 | 0.99 | 1.00 | **1.00** |
| Entropy | 0.99 | 0.99 | 0.99 | 0.99 | 0.99 | **0.99** |
| Kurtosis | 0.93 | 0.94 | 0.93 | 0.92 | 0.98 | **0.95** |
| Mean absolute Deviation | 0.98 | 0.99 | 0.99 | 0.99 | 1.00 | **1.00** |
| Median | 0.84 | 0.88 | 0.85 | 0.86 | 0.99 | **0.96** |
| Minimum | 0.39 | 0.65 | 0.66 | 0.48 | 0.46 | **0.67** |
| Range | 0.99 | 0.99 | 1.00 | 0.99 | 0.99 | **1.00** |
| Root Mean Square | 0.95 | 0.97 | 0.96 | 0.96 | 1.00 | **0.99** |
| Skewness | 0.90 | 0.92 | 0.91 | 0.90 | 0.97 | **0.92** |
| Standard Deviation | 0.99 | 0.99 | 0.99 | 0.99 | 1.00 | **1.00** |
| Uniformity | 0.96 | 0.95 | 0.95 | 0.96 | 0.97 | **0.95** |
| Variance | 0.98 | 0.99 | 0.98 | 0.98 | 0.99 | **1.00** |
| Coefficient of Variation | 0.81 | 0.84 | 0.83 | 0.83 | 0.98 | **0.94** |
| Surface Area | 0.97 | 0.95 | 0.97 | 0.95 | 0.99 | **0.98** |
| Compactness | 0.94 | 0.94 | 0.94 | 0.94 | 0.99 | **0.97** |
| Spherical disproportion | 0.95 | 0.98 | 0.95 | 0.98 | 0.99 | **0.97** |
| Sphericity | 0.98 | 0.98 | 0.97 | 0.98 | 0.99 | **0.99** |
| Surface to volume ratio | 0.91 | 0.90 | 0.90 | 0.89 | 0.98 | **0.90** |
| MeanDist | 0.99 | 0.98 | 0.99 | 0.98 | 0.99 | **0.99** |
| MaxDist | 1.00 | 1.00 | 1.00 | 1.00 | 1.00 | **1.00** |
| MeanDist_SUVmax_ | 0.98 | 0.98 | 0.98 | 0.98 | 0.98 | **0.99** |
| MaxDist_SUVmax_ | 0.99 | 1.00 | 0.99 | 1.00 | 1.00 | **1.00** |
| MeanDist_centroid_ | 0.98 | 0.98 | 0.98 | 0.98 | 0.98 | **0.99** |
| MaxDist_centroid_ | 0.99 | 1.00 | 0.99 | 1.00 | 1.00 | **1.00** |
| MeanDist_TLGmax_ | 0.97 | 0.98 | 0.98 | 0.98 | 0.98 | **0.97** |
| MaxDist_TLGmax_ | 0.99 | 1.00 | 1.00 | 0.99 | 1.00 | **1.00** |

**Table 6** Intraclass correlation coefficients (ICC) between features of patients’ tumor burden extracted from different segmentations: manual and semiautomatic using the standard Bayesian clustering (Bayes.).

| Features | **Standard Bayesian segmentation-related agreement** | | | | | |
| --- | --- | --- | --- | --- | --- | --- |
|  | Intra-observer | | Inter-observer | | | |
|  | Manual(Obs1)  vs  Bayes.(Obs1) | Manual(Obs2) vs  Bayes.(Obs2) | Manual(Obs1) vs  Bayes.(Obs2) | Manual(Obs2) vs  Bayes.(Obs1) | Bayes.(Obs1)  vs  Bayes.(Obs2) | Manual(Obs1)  vs  Manual(Obs2) |
| SUV_max_ | 1.00 | 1.00 | 1.00 | 1.00 | 1.00 | **1.00** |
| SUV_peak_ | 1.00 | 1.00 | 1.00 | 1.00 | 1.00 | **1.00** |
| SUV_mean_ | 0.86 | 0.90 | 0.88 | 0.88 | 0.99 | **0.98** |
| MTV | 0.91 | 0.89 | 0.92 | 0.89 | 0.99 | **0.97** |
| TLG | 0.97 | 0.97 | 0.98 | 0.97 | 1.00 | **1.00** |
| Energy | 0.99 | 0.99 | 0.99 | 0.99 | 0.99 | **1.00** |
| Entropy | 0.98 | 0.98 | 0.98 | 0.98 | 0.99 | **0.99** |
| Kurtosis | 0.91 | 0.91 | 0.94 | 0.87 | 0.98 | **0.95** |
| Mean absolute Deviation | 0.97 | 0.98 | 0.98 | 0.97 | 0.99 | **1.00** |
| Median | 0.76 | 0.82 | 0.78 | 0.79 | 0.99 | **0.96** |
| Minimum | 0.34 | 0.56 | 0.53 | 0.40 | 0.66 | **0.67** |
| Range | 0.98 | 0.99 | 0.99 | 0.98 | 0.99 | **1.00** |
| Root Mean Square | 0.92 | 0.95 | 0.94 | 0.93 | 0.99 | **0.99** |
| Skewness | 0.85 | 0.85 | 0.86 | 0.83 | 0.97 | **0.92** |
| Standard Deviation | 0.97 | 0.98 | 0.98 | 0.98 | 0.99 | **1.00** |
| Uniformity | 0.97 | 0.93 | 0.98 | 0.92 | 0.99 | **0.95** |
| Variance | 0.95 | 0.97 | 0.96 | 0.95 | 0.99 | **1.00** |
| Coefficient of Variation | 0.69 | 0.75 | 0.73 | 0.71 | 0.96 | **0.94** |
| Surface Area | 0.95 | 0.94 | 0.96 | 0.94 | 0.99 | **0.98** |
| Compactness | 0.88 | 0.90 | 0.90 | 0.89 | 0.99 | **0.97** |
| Spherical disproportion | 0.93 | 0.96 | 0.94 | 0.95 | 0.99 | **0.97** |
| Sphericity | 0.97 | 0.97 | 0.96 | 0.97 | 0.99 | **0.99** |
| Surface to volume ratio | 0.83 | 0.81 | 0.84 | 0.80 | 0.98 | **0.90** |
| MeanDist | 0.98 | 0.97 | 0.98 | 0.96 | 0.99 | **0.99** |
| MaxDist | 1.00 | 1.00 | 1.00 | 1.00 | 1.00 | **1.00** |
| MeanDist_SUVmax_ | 0.98 | 0.97 | 0.97 | 0.98 | 0.98 | **0.99** |
| MaxDist_SUVmax_ | 0.99 | 0.99 | 0.99 | 0.99 | 0.99 | **1.00** |
| MeanDist_centroid_ | 0.98 | 0.98 | 0.97 | 0.98 | 0.98 | **0.99** |
| MaxDist_centroid_ | 0.99 | 1.00 | 1.00 | 0.99 | 1.00 | **1.00** |
| MeanDist_TLGmax_ | 0.96 | 0.98 | 0.97 | 0.98 | 0.98 | **0.97** |
| MaxDist_TLGmax_ | 0.99 | 1.00 | 0.99 | 1.00 | 1.00 | **1.00** |

**Table 7** Intraclass correlation coefficients (ICC) between features of patients’ tumor burden extracted from different segmentations: manual and semiautomatic using absolute threshold (AT).

| Features | **Absolute threshold segmentation-related agreement** | | | | | |
| --- | --- | --- | --- | --- | --- | --- |
|  | Intra-observer | | Inter-observer | | | |
|  | Manual(Obs1)  vs  AT(Obs1) | Manual(Obs2) vs  AT(Obs2) | Manual(Obs1) vs  AT(Obs2) | Manual(Obs2) vs  AT(Obs1) | AT(Obs1)  vs  AT(Obs2) | Manual(Obs1)  vs  Manual(Obs2) |
| SUV_max_ | 1.00 | 1.00 | 1.00 | 1.00 | 1.00 | **1.00** |
| SUV_peak_ | 1.00 | 1.00 | 1.00 | 1.00 | 1.00 | **1.00** |
| SUV_mean_ | 0.76 | 0.66 | 0.70 | 0.72 | 0.98 | **0.98** |
| MTV | 0.83 | 0.76 | 0.79 | 0.80 | 0.99 | **0.97** |
| TLG | 0.98 | 0.97 | 0.97 | 0.97 | 0.99 | **1.00** |
| Energy | 1.00 | 0.99 | 1.00 | 0.99 | 1.00 | **1.00** |
| Entropy | 0.94 | 0.90 | 0.91 | 0.92 | 0.98 | **0.99** |
| Kurtosis | 0.77 | 0.65 | 0.73 | 0.68 | 0.97 | **0.95** |
| Mean absolute Deviation | 0.97 | 0.96 | 0.97 | 0.97 | 0.99 | **1.00** |
| Median | 0.46 | 0.42 | 0.35 | 0.33 | 0.93 | **0.96** |
| Minimum | 0.01 | 0.01 | 0.01 | 0.01 | 0.86 | **0.67** |
| Range | 0.99 | 0.99 | 0.99 | 0.99 | 1.00 | **1.00** |
| Root Mean Square | 0.89 | 0.83 | 0.86 | 0.86 | 0.99 | **0.99** |
| Skewness | 0.74 | 0.59 | 0.67 | 0.64 | 0.96 | **0.92** |
| Standard Deviation | 0.99 | 0.98 | 0.98 | 0.98 | 1.00 | **1.00** |
| Uniformity | 0.85 | 0.71 | 0.81 | 0.73 | 0.96 | **0.95** |
| Variance | 0.98 | 0.97 | 0.98 | 0.98 | 1.00 | **1.00** |
| Coefficient of Variation | 0.65 | 0.57 | 0.60 | 0.60 | 0.99 | **0.94** |
| Surface Area | 0.91 | 0.83 | 0.87 | 0.87 | 0.98 | **0.98** |
| Compactness | 0.81 | 0.80 | 0.80 | 0.80 | 0.99 | **0.97** |
| Spherical disproportion | 0.90 | 0.94 | 0.91 | 0.94 | 0.98 | **0.97** |
| Sphericity | 0.95 | 0.93 | 0.95 | 0.93 | 0.98 | **0.99** |
| Surface to volume ratio | 0.62 | 0.53 | 0.60 | 0.52 | 0.98 | **0.90** |
| MeanDist | 0.98 | 0.98 | 0.98 | 0.97 | 0.99 | **0.99** |
| MaxDist | 1.00 | 1.00 | 1.00 | 1.00 | 1.00 | **1.00** |
| MeanDist_SUVmax_ | 0.96 | 0.96 | 0.96 | 0.96 | 0.98 | **0.99** |
| MaxDist_SUVmax_ | 0.99 | 0.99 | 0.99 | 0.99 | 1.00 | **1.00** |
| MeanDist_centroid_ | 0.96 | 0.96 | 0.96 | 0.97 | 0.98 | **0.99** |
| MaxDist_centroid_ | 0.99 | 1.00 | 0.99 | 0.99 | 1.00 | **1.00** |
| MeanDist_TLGmax_ | 0.97 | 0.96 | 0.96 | 0.96 | 0.98 | **0.97** |
| MaxDist_TLGmax_ | 0.99 | 1.00 | 1.00 | 1.00 | 1.00 | **1.00** |

**Table 8** Intraclass correlation coefficients (ICC) between features of patients’ tumor burden extracted from different segmentations: manual and semiautomatic using relative threshold (RT).

| Features | **Relative threshold segmentation-related agreement** | | | | | |
| --- | --- | --- | --- | --- | --- | --- |
|  | Intra-observer | | Inter-observer | | | |
|  | Manual(Obs1)  vs  RT(Obs1) | Manual(Obs2) vs  RT(Obs2) | Manual(Obs1) vs  RT(Obs2) | Manual(Obs2) vs  RT(Obs1) | RT(Obs1)  vs  RT(Obs2) | Manual(Obs1)  vs  Manual(Obs2) |
| SUV_max_ | 1.00 | 1.00 | 1.00 | 1.00 | 1.00 | **1.00** |
| SUV_peak_ | 1.00 | 1.00 | 1.00 | 1.00 | 1.00 | **1.00** |
| SUV_mean_ | 0.72 | 0.75 | 0.73 | 0.75 | 1.00 | **0.98** |
| MTV | 0.66 | 0.63 | 0.66 | 0.64 | 0.97 | **0.97** |
| TLG | 0.72 | 0.72 | 0.72 | 0.72 | 0.99 | **1.00** |
| Energy | 0.89 | 0.89 | 0.89 | 0.89 | 1.00 | **1.00** |
| Entropy | 0.95 | 0.95 | 0.95 | 0.94 | 0.99 | **0.99** |
| Kurtosis | 0.74 | 0.65 | 0.68 | 0.73 | 0.94 | **0.95** |
| Mean absolute Deviation | 0.87 | 0.88 | 0.87 | 0.87 | 1.00 | **1.00** |
| Median | 0.59 | 0.62 | 0.59 | 0.62 | 1.00 | **0.96** |
| Minimum | 0.16 | 0.33 | 0.30 | 0.20 | 0.63 | **0.67** |
| Range | 0.95 | 0.98 | 0.98 | 0.95 | 0.97 | **1.00** |
| Root Mean Square | 0.82 | 0.84 | 0.82 | 0.84 | 1.00 | **0.99** |
| Skewness | 0.51 | 0.46 | 0.48 | 0.50 | 0.91 | **0.92** |
| Standard Deviation | 0.89 | 0.90 | 0.90 | 0.89 | 0.99 | **1.00** |
| Uniformity | 0.91 | 0.84 | 0.91 | 0.84 | 0.96 | **0.95** |
| Variance | 0.78 | 0.82 | 0.81 | 0.79 | 0.99 | **1.00** |
| Coefficient of Variation | 0.43 | 0.39 | 0.42 | 0.43 | 0.96 | **0.94** |
| Surface Area | 0.80 | 0.81 | 0.81 | 0.81 | 0.99 | **0.98** |
| Compactness | 0.59 | 0.60 | 0.60 | 0.61 | 0.93 | **0.97** |
| Spherical disproportion | 0.88 | 0.87 | 0.91 | 0.90 | 0.93 | **0.97** |
| Sphericity | 0.93 | 0.93 | 0.94 | 0.93 | 0.98 | **0.99** |
| Surface to volume ratio | 0.41 | 0.42 | 0.44 | 0.44 | 0.87 | **0.90** |
| MeanDist | 0.96 | 0.94 | 0.96 | 0.93 | 0.99 | **0.99** |
| MaxDist | 0.99 | 1.00 | 0.99 | 1.00 | 1.00 | **1.00** |
| MeanDist_SUVmax_ | 0.96 | 0.97 | 0.96 | 0.96 | 0.99 | **0.99** |
| MaxDist_SUVmax_ | 0.99 | 0.99 | 0.99 | 0.99 | 0.99 | **1.00** |
| MeanDist_centroid_ | 0.95 | 0.97 | 0.97 | 0.96 | 0.98 | **0.99** |
| MaxDist_centroid_ | 0.99 | 0.99 | 0.99 | 0.99 | 1.00 | **1.00** |
| MeanDist_TLGmax_ | 0.94 | 0.96 | 0.95 | 0.96 | 0.99 | **0.97** |
| MaxDist_TLGmax_ | 0.99 | 1.00 | 0.99 | 1.00 | 1.00 | **1.00** |
